# Smoking significantly impairs clinical outcome following anterior cervical radiculopathy surgery

**DOI:** 10.1016/j.bas.2026.106030

**Published:** 2026-04-01

**Authors:** Azra Gül, Daniela Limbania, Caroline MW. Goedmakers, Timothy R. Smith, Carmen Vleggeert-Lankamp Lankamp

**Affiliations:** a Department of Neurosurgery, Leiden University Medical Centre, Leiden, the Netherlands; b Computational Neuroscience Outcomes Center, Department of Neurosurgery, Brigham and Women's Hospital, Harvard Medical School, Boston, MA, USA; c Department of Neurosurgery, Boston Medical Center, Boston, MA, USA; d Department of Neurosurgery, Spaarne Gasthuis, Hoofddorp, Haarlem, the Netherlands

**Keywords:** ACDF, Cervical radiculopathy, Smoking, VAS, Fusion

## Abstract

**Introduction:**

Smoking impairs bone healing and vascularization, which may adversely affect recovery after spinal instrumented spondylodesis surgery. However, the effects of smoking on the clinical outcomes after anterior surgery for cervical radiculopathy remain unclear.

**Research questions:**

(1) Is smoking associated with clinical outcomes after single-level ACDF?

(2) Does smoking affect spinal fusion/stability?

**Methods:**

This retrospective cohort study included 482 patients who underwent single-level ACDF between May 2029 and November 2024, comprising 202 smokers and 208 non-smokers. The Visual Analogue Scale arm and neck pain, pain interference, pain intensity, physical function and the fusion/stability served as outcome measures. Data was collected at baseline, 3-, 6-, 12- and 24 months post-surgery and analysed using linear mixed-effects models. In addition, to evaluate outcomes at the level of individual success, predefined cut-off values were applied (arm pain ≤2.5, neck pain ≤3.5). The presence of spinal fusion was assessed on dynamic radiographs 6- and 12 months post surgery.

**Results:**

Baseline characteristics were comparable. Over 24 months, smokers reported 1.65 points higher arm pain (p < 0.001) and 1.56 points higher neck pain (p = 0.003) compared to non-smokers. Moreover, at 24 months, non-smokers achieved higher successful outcomes for arm pain (71.0% vs 52.3%, p = 0.043), but not for neck pain (73.1% vs 60%, p = 0.158). Fusion/stability rates were similar at 6 months (56.1% versus 57.1%, p = 0.943), but after 12 months, non-smokers demonstrated a higher fusion/stability rate (71.7% vs 51.4%, p = 0.006).

**Discussion and conclusion:**

Non-smokers achieve both better long-term clinical outcomes and higher fusion/stability rates compared to smokers in ACDF surgery.

## Highlights

• Smokers report higher arm pain one and two years after single-level ACDF.
• Non-smokers achieve higher spinal fusion rates at 12 months follow-up.
• Smoking status is associated with less favourable clinical outcomes.
• Smoking status may be relevant in preoperative evaluation for ACDF.

## 1 Introduction

Cigarette smoking rates have decreased substantially over the past five decades, yet tobacco use continues to pose a significant health concern for health-care providers worldwide, with an estimated one in five adults consuming tobacco (American Lung Association; World Health Organization, 2024). Comparative analyses indicate that the risk of all-cause mortality in smokers is more than double that of never smokers in both men and women (Lariscy et al., 2018). In the context of spine surgery, smoking has been associated with impaired bone healing, primarily attributable to its deleterious effects on bone metabolism (Yoon et al., 2012; Chen et al., 2011; Williams and Moseley, 2013; Cusano, 2015). Additionally, multiple studies have demonstrated that smoking is linked to higher complication rates, reduced fusion success, and poorer clinical outcomes in patients undergoing spine surgery (Andersen et al., 2001; Bisson et al., 2015; De la Garza Ramos et al., 2017; Elsamadicy et al., 2017; Hermann et al., 2016a; Seicean et al., 2013). However, the impact of smoking on clinical outcomes in patients with cervical radiculopathy undergoing anterior surgical intervention is yet to be elucidated.

The most commonly performed surgical intervention for patients with cervical radiculopathy, caused by intervertebral disc herniation, is an Anterior Cervical Discectomy and Fusion (ACDF), utilizing an anterior approach to decompress the affected spinal nerve root through discectomy. The objective of ACDF is to remove the damaged disc and interconnect the adjacent vertebrae and thereby facilitate fusion. The detrimental effect of smoking on fusion rates was described previously (Hilibrand et al., 2001a; Peolsson et al., 2006; Pearson et al., 2016), without however making the correlation to clinical conditions.

The aim of the present study is to investigate the association between clinical outcomes in smokers and non-smokers who underwent a single-level ACDF. In addition, radiographic fusion/stability status at 6- and 12 months postoperatively will be assessed, using the de Vries-Vleggeert parameter (de Vries et al., 2024) to determine whether differences in fusion rates between smokers and non-smokers correspond to differences in clinical improvement.

## 2 Material and methods

### 2.1 Study design and setting

This retrospective cohort study was conducted using data from the Brigham and Woman's Hospital in the United States from May 2019 to November 2024. The study adheres to The Strengthening the Reporting of Observational Studies in Epidemiology (STROBE) guidelines (von Elm et al., 2007). Approval was obtained from the institutional review board and informed consent was waived due to the retrospective design of the study. The study protocol was approved by the Institutional Review Board (IRB) of the BWH (IRB no 2015P002352). Patients that were operated in Boston routinely complete patient-reported outcome measurement questionnaires as part of standard postoperative care and follow-up which served as outcome parameters. Patients received standard PROMs questionnaires by mail at baseline, 3, 6, 12 and 24 months after surgery.

### 2.2 Eligibility

The study population comprised of patients diagnosed with cervical radiculopathy who underwent single-level ACDF from whom recent smoking behaviour was known at the day of surgery and who had a minimum of six months of clinical and radiological follow-up available. Exclusion criteria included patients with a history of prior spine surgery or those who underwent surgery for cervical malignancy.

### 2.3 Baseline characteristics

Baseline characteristics including age, gender, body mass index (BMI), smoking behaviour, amount of pack years, anxiety, -depression (Patient-Reported Outcome Measures) and alcohol consumption (AUDIT-C) were retrieved. Assessment of smoking behaviour was evaluated based on patient notes written by either a medical doctor or physician assistant prior to surgery and throughout the entire follow-up period. Anxiety/depression offers insights into the patient's overall functioning through the PROMIS Anxiety and Depression item banks ranging from 41.0 to 79.4 with higher scores indicating a higher impact on daily life (Health Measures, 2025; Health, 2025).

### 2.4 Clinical outcomes

The clinical outcome analysis is focused on assessing the patient reported outcomes for arm pain, neck pain, pain interference, pain intensity and physical function. Questionnaires were sent to the patients at baseline, 3-, 6-, 12- and 24-months after surgical intervention. Arm pain and neck pain are evaluated with the Visual Analogue Scale (VAS), which ranges from 0 (no pain) to 10 (worst pain imaginable) and is known for its reliability and validity (Huskisson, 1974; Carlsson, 1983).

To assess the pain interference, pain intensity and physical function, the Patient Reported Outcomes Measurement Information System (PROMIS) instruments were used. The range from a PROMIS outcome is calculated through item response theory and expressed as a standardized T-score, centred around the average of the general population, rather than a 0-100 scale. The exact range depends on factors such as the specific PROMIS domain and the number and type of items used in the short form. Therefore, the lowest possible score may not be 0, but is determined by the statistical properties of the item bank.

The pain interference item bank reflects the extent to which the experienced pain interferes with daily life activities on a scale of 41.6 to 75.6, where a higher score reflects more interference (https://www.healthmeasures.net/images/PROMIS/manuals/PROMIS_Pain_Interference_Scoring_Manual.pdf.). Pain intensity provides an understanding of the level of pain a patient is enduring on a scale of 30 (no pain) to 81.8 (most intense pain imageable) (https://www.healthmeasures.net/images/PROMIS/manuals/PROMIS_Pain_Intensity_Scoring_Manual.pdf.). The physical function item bank illustrates self-reported capability in daily life on a scale of 13.5 until 61.9, a higher score corresponds with a better physical function (https://www.healthmeasures.net/images/PROMIS/manuals/PROMIS_Physical_Function_Scoring_Manual.pdf.).

Another evaluation method is to dichotomize outcome data to evaluate the percentage of patients that reports a ‘successful outcome’, using pre-published cut off values for ‘success’. The following cut off values were used and considered as a ‘successful outcome’: VAS Arm pain ≤2.5 and VAS neck pain ≤3.5 (Mjåset et al., 2020).

### 2.5 Radiological evaluation

**Fig. 1:**
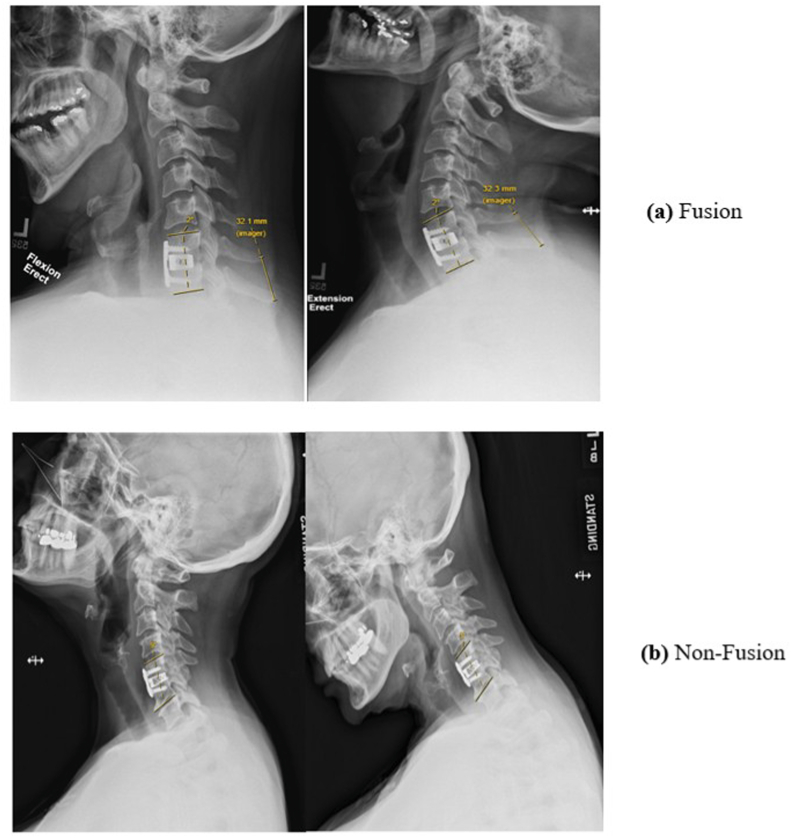
**Demonstration of Fusion Assessment: (a) Fusion** target level demonstrated less than 3.0° difference in Cobb angle and less than 2.0 mm difference in interspinous distance comparing flexion and extension radiographs. (b) the two required conditions were not fulfilled.

Radiological follow-up was conducted at 6- and 12 months after surgery using dynamic radiographs of the cervical spine. Fusion/stability was established by evaluating stability by applying the de Vries-Vleggeert parameter: a combination of the Cobb angle and the interspinous distance of the target level assessed on flexion extension radiographs (Fig. 1). Two researchers (AG, DL) independently assessed spinal fusion/stability. Agreement was reached through group discussion involving the senior neurosurgeon (CVL). Cut off values are defined as; Cobb angle difference at the target level is < 3.0° and the interspinous distance difference <2.0 mm (de Vries et al., 2024). To assess the reliability of the grading system, a subset of flexion extension radiographs was re-scored to evaluate both inter- and intra-rater agreement, yielding satisfactory consistency (Appendix 1).

### 2.6 Statistical analysis

Normally distributed variables are presented as mean ± standard deviation (SD). Differences between smokers and non-smokers were analysed using linear mixed-effects models including a random intercept for each subject to account for repeated measures. In longitudinal datasets, missing data is inevitable. Furthermore, multiple imputation may introduce instability in mixed-model analysis (Twisk et al., 2013). Thereby, linear mixed-effects are well-suited for handling longitudinal data thus the application of multiple imputation was deemed unnecessary. The linear mixed-model analysis was adjusted for baseline differences, age, gender, BMI and anxiety, -depression. To compare the clinical success percentages and the fusion percentages Pearson Chi-Squared tests are utilized, additional sensitivity analyses using the Fisher's exact test were performed to confirm chi-squared results. A p-value of 0.05 or lower was considered statistically significant. IBM SPSS software, version 30.0, was used for all statistical analysis.

## 3 Results

### 3.1 Demographics, description of study population

A total of 1294 patients who underwent an ACDF procedure at the Brigham and Woman's Hospital were identified from a retrospective database between May 2019 and November 2024. These patients were part of an existing database in which data was collected at baseline and during follow-up as part of routine clinical care. To ensure that the potential risk for selection bias is minimized, all consecutive patients who met the inclusion criteria were included in the database and appropriate data were retrieved for the study. Of the initial cohort, 460 were excluded for having the diagnosis cervical myelopathy as opposed to cervical radiculopathy, and another 300 as they underwent multi-level ACDF. An additional 5 patients were excluded due to prior surgery and another 2 because they underwent surgery for cervical malignancy. Finally, 45 patients were excluded due to insufficient clinical outcome follow-up, which was defined as the absence of any clinical outcome at any of the follow-up points. Thus, a total of 482 patients with cervical radiculopathy that underwent a single-level ACDF procedure from the BWH were included in the study, from which 414 patients received a titanium cage with a plate, 54 patients received a PEEK cage with integrated screws and 14 patients received a titanium standalone cage.

280 patients were non-smokers and 202 patients belonged to the smokers group. All other baseline characteristics were comparable between the non-smokers and smokers (Table 1).

**Table 1:** Baseline demographics.

Table 1; Patient Demographics; baseline demographics of all patients. The groups are comparable at baseline.		
	Non-smokers (n = 280)	Smokers (n = 202)
**Pack Years, mean, SD**^a^	0	18.6 ± 17.8
**Male (%)**	153 (44)	100 (46)
**Mean Age, yrs (SD)**	55.3 ± 14	58.2 ± 13
**Body Mass Index kg/m^2^ (SD)**	28.8 ± 6	28.6 ± 5
**Anxiety-Depression (SD)**	50.5 ± 9	52.9 ± 9
**Pain Interference (SD)**	63.4 ± 9	65.5 ± 7
**Pain Intensity (SD)**	55.8 ± 9	57.3 ± 8
**Physical Function (SD)**	39.7 ± 9	36.6 ± 7
**Arm Pain (SD)**	6.2 ± 2.6	5.9 ± 2.4
**Neck Pain (SD)**	5.5 ± 2.8	5.9 ± 2.7
**Audit-C (SD)**	3.4 ± 6.0	2.8 ± 5.5

a SD; Standard Deviation.

### 3.2 Clinical outcomes in non-smokers versus smokers

All outcome measures improved after surgery.

#### 3.2.1 VAS arm pain

The non-smokers demonstrated a more pronounced reduction in VAS arm pain one year after surgery from 6.2 ± 3 to 2.3 ± 3 compared to 5.9 ± 2 to 3.1 ± 2 in the smoker's group (p = 0.023). At the two-year follow-up timepoint, arm pain further decreased to 1.9 ± 2 in the non-smoking group while smokers reported a similar mean score of 3.5 ± 3 (p < 0.001) (Table 2). In addition, a linear mixed-effects model, correcting for baseline differences in arm pain, age, gender, Body Mass Index (BMI) and anxiety,-/depression showed that smoking was associated with higher arm pain scores after one and two years compared to non-smokers, with smokers experiencing an average of 0.990 higher arm pain score over the course of one year (p = 0.012) and a 1.650 higher score over the course of two years following (p < 0.001) (Table 3). The proportion of individual clinical arm pain success defined by pre-established cut-off values was similar after one year when comparing non-smokers to smokers 59.8% (95% CI: 53.8 to 65.7%) versus 53.1% (95% CI: 46.2 to 60.0%, p = 0.379). After two years however, the non-smokers demonstrated a significant higher success percentage 71.0 % (95% CI: 65.5 to 76.5%) versus 52.3% (95% CI: 45.4 to 59.2%, p = 0.043). Notably, no difference in success percentage was observed between patients who smoked less or more than one pack per day (Table 4).

**Table 2:** Mean values.

Table 2: Mean values of clinical outcomes at baseline, 3, 6, 12 and 24 months of follow-up with standard deviations. P-values for the between group comparisons are given for each time point comparing the non-smokers with the smokers.				
	Time (months)	Non-Smokers (n = 280)	Smokers (n = 202)	p
**Arm pain**	**0**	6.2 ± 3	5.9 ± 2	0.293
	**3**	3.2 ± 3	2.7 ± 3	0.191
	**6**	2.4 ± 3	2.7 ± 2	0.572
	**12**	2.3 ± 3	3.1 ± 2	0.023
	**24**	1.9 ± 2	3.5 ± 3	<0.001

**Neck pain**	**0**	5.5 ± 3	5.9 ± 3	0.534
	**3**	3.9 ± 3	3.7 ± 3	0.742
	**6**	3.0 ± 3	3.2 ± 3	0.565
	**12**	2.8 ± 3	3.7 ± 3	0.034
	**24**	2.7 ± 3	3.8 ± 3	0.027
**Pain Interference**	**0**	63.4 ± 9	66.0 ± 7	0.085
	**3**	57.2 ± 9	58.3 ± 11	0.245
	**6**	55.2 ± 10	60.5 ± 10	0.207
	**12**	56.4 ± 10	59.6 ± 9	0.349
	**24**	53.7 ± 9	60.7 ± 7	0.092

**PainIntensity**	**0**	55.8 ± 9	57.8 ± 8	0.368
	**3**	50.3 ± 9	49.8 ± 10	0.866
	**6**	50.5 ± 9	50.8 ± 9	0.952
	**12**	50.2 ± 10	50.4 ± 8	0.499
**Physical Function**	**24**	51.6 ± 11	58.8 ± 9	0.096
	**0**	39.7 ± 9	36.6 ± 7	0.469
	**3**	41.1 ± 8	39.2 ± 7	0.884
	**6**	47.7 ± 8	40.6 ± 8	0.234
	**12**	43.6 ± 10	38.3 ± 7	0.192
	**24**	45.2 ± 10	41.4 ± 9	0.322

**Table 3:** Linear mixed-effects models.

Table 3: Linear mixed-effects models including random intercepts, ∗adjusted for baseline differences, Body Mass Index, Age, Gender and Axiety-/Depression				
	**Time**	**B∗**	**95% CI**	***p***
**Arm pain**	Overall	0.469	−0.04; 0.98	0.073
	3	−0.297	−1.00; 0.41	0.412
	6	0.464	−0.24; 1.17	0.198
	12	0.990	0.22; 1.77	0.012
	24	1.650	0.69; 2.62	<0.001

**NECK PAIN**	Overall	0.165	−0.39; 0.72	0.557
	3	−0.289	−1.00; 0.43	0.430
	6	−0.065	−0.78; 0.65	0.859
	12	0.674	−0.14; 1.48	0.102
	24	1.559	0.53; 2.59	0.003
**Pain Interference**	Overall	1.644	−0.05; 3.34	0.058
	3	0.722	−1.47; 2.92	0.519
	6	2.680	0.46; 4.90	0.018
	12	1.191	−1.33; 3.71	0.353
	24	3.333	0.32; 6.34	0.030
**Pain intensity**	Overall	0.709	−1.14; 2.56	0.452
	3	0.756	−1.62; 3.13	0.532
	6	−0.513	−2.94; 1.91	0.678
	12	0.188	−2.53; 2.91	0.892
	24	0.709	−1.14; 2.56	0.425
**Physical Function**	Overall	−1.025	−2.65; 0.60	0.216
	3	−1.242	−3.35; 0.87	0.248
	6	−1.091	−3.17; 0.98	0.301
	12	−1.823	−4.12; 0.47	0.119
	24	−0.841	−3.41; 1.73	0.521

**Table 4:** Percentage of patients with a successful outcome based on pre-established cut-off values for Patient-Reported Arm and Neck pain (0-10) success in three groups. Additionally, the 95% Confidence Intervals are reported. Differences between non-smokers and smokers (merge) were tested with a Pearson's chi-squared test.

	**Time**	Arm pain	Arm pain	Arm pain	Arm pain	p
		≤2.5	≤2.5	≤2.5	≤2.5	
		Non-smokers	Smoking <1 pack/day	Smoking >1 pack/day	Smokers (merge)	
**Arm Pain ≤2.5**	**0 Months**	10.8% (7.3; 14.3%)	12.5% (5.7; 19.3%)	8.5% (2.9; 14.1%)	10.4% (6.4; 14.4%)	0.909
	**3 Months**	51.2% (45.2; 57.2%)	57.5% (47.2; 67.8%)	57.8% (47.3; 68.3%)	58.1% (51.3; 64.9%)	0.295
	**6 Months**	60.9% (55.0; 66.8%)	59.5% (49.2; 69.8%)	51.1% (40.4; 61.8%)	54.4% (47.6; 61.2%)	0.323
	**12 Months**	59.8% (53.8; 65.7%)	56.0% (45.6; 66.4%)	51.3% (40.6; 62.0%)	53.1% (46.2; 60.0%)	0.379
	**24 Months**	71.0% (65.5; 76.5%)	58.8% (48.5; 69.1%)	48.1% (37.5; 58.7%)	52.3% (45.4; 59.2%)	0.043

**Neck Pain ≤3.5**	**0 Months**	28.5% (23.2; 33.8%)	22.1% (13.6; 30.6%)	23.2% (14.2; 32.2%)	22.5% (16.8; 28.2%)	0.173
	**3 Months**	49.4% (43.4; 55.4%)	52.6% (42.3; 62.9%)	51.1% (40.4; 61.8%)	52.3% (45.4; 59.2%)	0.660
	**6 Months**	62.5% (56.7; 68.3%)	60.5% (50.2; 70.8%)	53.3% (42.6; 64.0%)	57.3% (50.5; 64.1%)	0.421
	**12 Months**	67.2% (61.6; 72.8%)	56.0% (45.6; 66.4%)	55.0% (44.3; 65.7%)	55.4% (48.6; 62.2%)	0.110
	**24 Months**	73.1% (67.7; 78.5%)	56.3% (45.9; 66.7%)	62.5% (52.0; 73.0%)	60.0% (53.3; 66.7%)	0.158

#### 3.2.2 VAS neck pain

The trajectory of neck pain, as measured by the VAS neck pain, mirrored that of arm pain, with non-smokers reporting significantly lower VAS neck pain scores at both one and two years postoperatively compared to smokers:2.8 ± 3 versus 3.7 ± 3 (p = 0.034) at one year, and 2.7 ± 3 versus 3.8 ± 3 (0.027) at two years (Table 2). Although linear mixed-effects modelling did not demonstrate significant differences at the one-year follow-up, smokers experienced a significant increase of 1.559 points in neck pain over two years relative to non-smokers (p = 0.003) (Table 3). At one year postoperatively, the rate of clinical success was 67.2% (95% CI: 61.6% to 72.8%) in the non-smoking cohort compared with 55.4% (95% CI: 48.6 to 62.2%) in the smoking cohort (p = 0.110). At two years, clinical success rates were 73.1% (95% CI: 67.7 to 78.5%) in non-smokers and 60.0% (95% CI: 53.3 to 66.7%) in smokers (p = 0.158).

#### 3.2.3 Pain interference

After one year follow-up, the non-smokers reported a mean pain interference of 56.4 ± 10 compared to 59.6 ± 9 in the smokers (p = 0.349). After two years, a more pronounced difference can be observed with 53.7 ± 9 in the non-smokers versus 60.7 ± 7 in the smoker's group (p = 0.092) (Table 2). Likewise, the linear mixed-effects models showed no difference between the groups after one year (p = 0.353), but after two years, the smokers reported a 3.333-point higher pain interference (p = 0.030) (Table 3).

#### 3.2.4 Pain intensity and physical function

Pain intensity and physical function all improved equally in the smokers versus non-smokers. A mean difference of 0.188 (p = 0.892) and 0.709 (p = 0.425) after one and two years in pain intensity was shown by a linear mixed-effects model. This was 1.828 (p = 0.119) and 0.841 (p = 0.521) for the physical function after one and two years.

### 3.3 The influence of pack years

An additional linear regression analysis was conducted to assess the impact of the number of pack years on clinical outcomes, aiming to determine whether a higher number of pack years was correlated with worse clinical outcomes. This analysis however revealed no significant association between an increased number of pack years and worse clinical outcome (Appendix 2).

### 3.4 Spinal fusion/stability in non-smokers versus smokers

At six months, a total of 260 flexion extension (dynamic) radiographs were available: 140 from non-smokers and 120 from smokers. In the non-smoking group, 80 out of 140 patients achieved stability (‘fusion’) (57.1%) and 68 out of 120 in the smoking group achieved fusion (56.7%) (p = 0.943) After one year, 190 flexion-extension radiographs were available: 86 out of 120 achieved fusion (71.7%) compared to 36 out of 70 in the smokers (51.4%) (p = 0.006).

## 4 Discussion

Smoking is a common, modifiable risk factor that impairs bone healing and fusion, which are key for successful outcomes after cervical spine surgery. Its impact on patient-reported outcomes has been unclear, with prior studies limited by small cohorts and insufficient adjustment for confounders. The findings demonstrate that smoking significantly impairs clinical outcomes, with non-smokers experiencing less arm and neck pain both one and two years after surgery. Moreover, a significantly higher proportion of non-smokers achieve fusion in comparison to the smoking group after a time period of 12 months. These findings emphasize the relevance of smoking status in preoperative evaluation and underline the broader clinical implications of smoking on postoperative outcomes following ACDF.

### 4.1 Smoking and clinical outcomes after ACDF

Fusion has been reported to play a critical role for successful outcomes and clinical outcomes following cervical spine procedures (Bohlman et al., 1993; Bose, 2001; Cuathen et al., 1998; Farey et al., 1990). Previous literature focusing on the relationship between smoking and spinal fusion has consistently demonstrated a negative impact of smoking on fusion rates (Brown et al., 1986; Cerier et al., 2019; Hermann et al., 2016b; Hilibrand et al., 2001b; Lau et al., 2014). The underlying assumption has been that impaired fusion would translate into poorer clinical outcomes, particularly regarding arm pain, which is the primary indication for surgical treatment in contrast to prolonged conservative treatment. The findings in the current study corroborate with this hypothesis, showing that non-smokers experience significantly less arm pain compared to smokers. Notably, the non-smokers additionally reported less neck pain, an outcome that is not the main surgical target but remains highly relevant to patients’ overall well-being. This observation highlights that the detrimental effects of smoking extend beyond the primary surgical indication, emphasizing the broader clinical impact on patient recovery.

The studies that evaluated the relationship between smoking and postoperative pain outcomes in ACDF patients, demonstrated limited and often inconclusive findings (Mangan et al., 2021; Patel et al., 2019; Toci et al., 2022; Wang et al., 2021). Mangan et al. reported no significant effect of smoking on outcomes after multi-level ACDF, however, only 16% of patients were smokers, baseline differences were not adjusted for and potential confounders were not considered (Mangan et al., 2021). Similarly, Patel et al. and Toci et al. found no impact of smoking on pain outcomes, yet both studies included very small smoker cohorts (25 and 35 patients) leading to limited statistical power (Patel et al., 2019; Toci et al., 2022). Wang et al. evaluated multilevel hybrid cervical surgery and reported that current smokers exhibited poorer early fusion and lower 1-year fusion rates, although clinical outcomes were similar across groups (Wang et al., 2021). Previously, Cerier et al. (2019) reported that non-smokers exhibited better NDI scores, although their study was limited by a relatively small sample size of 61 patients. The present study builds upon these findings by additionally demonstrating improvements in both arm and neck pain, outcomes that are of clinical relevance to both surgeons and patients. Moreover, the current analysis benefits from a larger cohort with well-balanced baseline characteristics and statistical adjustment for potential confounders.

### 4.2 Smoking and spinal fusion

In the present study, no significant differences in fusion rates were observed between smokers and non-smokers at 6 months, however, at 12 months non-smokers exhibited higher rates of fusion. These findings are in line with previous studies demonstrating that smoking is associated with an elevated risk of pseudarthrosis and reduced fusion success following both cervical and lumbar procedures (Hermann et al., 2016b; Hilibrand et al., 2001b; Lau et al., 2014). The underlying pathophysiological mechanisms are multifactorial: nicotine and other toxic constituents of cigarette smoke impair angiogenesis and microvascular perfusion, inhibit osteoblast proliferation and differentiation, reduce bone matrix synthesis and mineralization, and promote a pro-inflammatory environment that increases susceptibility to infection. Collectively, these processes interfere with normal bone remodelling and consolidation, thereby compromising the establishment of a solid arthrodesis.

In this context, the comparable fusion rates at 6 months may be attributable to the timing of evaluation, as insufficient time may have elapsed for differences in bone consolidation to become apparent. While the present study assessed fusion at 6- and 12 months, prior studies that have reported differences evaluated fusion 12 months or later (Hermann et al., 2016b; Hilibrand et al., 2001b; Lau et al., 2014), suggesting that shorter follow-up intervals may underestimate the adverse effects of smoking on spinal fusion/stability.

While the detrimental effects of smoking on outcome of ACDF surgery are convincing with the data from the current study, the optimal duration of smoking cessation required to mitigate this risk remains unclear. Existing evidence provides limited guidance on whether cessation before or after surgery improves fusion outcomes, and no consensus has been reached regarding the minimum period of abstinence necessary to reverse the adverse biological effects of smoking. In our cohort, no significant association was observed between the number of pack years and clinical outcomes (Appendix 2), suggesting that cumulative exposure did not exert a measurable influence within the follow-up period.

### 4.3 Strengths and limitations

This retrospective study has its limitations. The exclusion of 45 patients due to the absence of clinical follow-up data, which may have been caused by factors such as loss to follow-up, relocation or seeking care elsewhere could potentially have affected the generalizability of the findings. Moreover, not all patients at 6- and 12-months follow-up had a flexion-extension radiograph available. Both could raise concerns about potential selection bias. However, an analysis comparing baseline characteristics of the 45 excluded patients to the 482 included patients showed no significant differences. The second limitation is ascribed to the clinical routine in which patients receive surgery at the Brigham and Women's Hospital, yet frequently receive follow-up care at external institutions, making it difficult to consistently obtain postoperative imaging. An analysis comparing baseline and clinical parameters between patients with and without follow-up radiographs showed no significant differences, suggesting that the absence of follow-op x-rays is unlikely to have introduced potential bias. Strengths of the study include being the first study with a large cohort to show the current results.

### 4.4 Generalizability of study results

The study cohort was derived from a single high-volume academic center and focused primarily on patients undergoing single-level ACDF. Differences in surgical techniques, perioperative care, and patient demographics across institutions and healthcare systems may influence outcomes. Nevertheless, the large sample size, adjustment for potential confounders, and consistency of the results with established biological mechanisms and prior literature support the external validity of the findings. Taken together, these factors suggest that the detrimental impact of smoking on both fusion/stability and clinical outcomes is likely applicable to broader ACDF populations, although replication in diverse clinical settings remains to be explored.

## 5 Conclusion

Smoking negatively affects clinical outcome after single-level ACDF in patients with cervical radiculopathy. These findings highlight the detrimental impact of smoking on postoperative recovery and spinal fusion. Given its association with less favourable clinical and radiographic outcomes, smoking status should be taken into account when counselling patients scheduled for ACDF. Patients should be informed preoperatively about the increased risk of suboptimal recovery after ACDF while smoking.

## Ethical review committee statement

Approval for the project entitled ‘Smoking Significantly Impairs Clinical Outcome Following Anterior Cervical Radiculopathy Surgery’ was obtained from the Institutional Review Board (IRB) of the Brigham and Women's Hospital, Boston, MA, USA(IRB no 2015P002352). The requirement for informed consent was waived due to the retrospective design of the study, which involved no direct contact with human or animal subjects.

## Funding

This research did not receive any specific grant from funding agencies in the public, commercial, or not-for-profit sectors.

## Declaration of competing interest

The authors declare that they have no known competing financial interests or personal relationships that could have appeared to influence the work reported in this paper.

## Data Availability

Data cannot be shared publicly because of ethical concerns. If a request is done, this can be reviewed by the Medical Ethical Committee of the LUMC, data can be shared.
