# Supplementary material for: Smoking significantly impairs clinical outcome following anterior cervical radiculopathy surgery
Source: Brain Spine. 2026 Apr 1;6:106030. doi: 10.1016/j.bas.2026.106030 (PMC13089042; doi:10.1016/j.bas.2026.106030)
Supplement: Multimedia component 1 [file mmc1.docx]

**Appendix 1: Inter- and Intra-Rater agreement**

Spinal fusion/stability is a dichotomous outcome defined by either fusion (1) or non-fusion (0). Both raters (AG, DL) independently classified all available radiographs at 6- and 12 months follow-up. Inter-rater agreement was quantified using Cohen’s kappa coefficient (κ), which accounts for agreement occurring by chance. For intra-rater reliability, one rater re-assessed a random subset of 50 radiographs after a four-week interval, blinded to their initial assessment, and κ was again calculated.


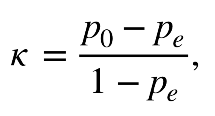
κ is calculated using IBM SPSS software, version 30.0 according to the following formula:

For the interpretation of κ, the guidelines of Landis & Koch were utilized (1);

<0.20 = slight agreement

0.21–0.40 = fair agreement

0.41–0.60 = moderate agreement

0.61–0.80 = substantial agreement

0.81–1.00 = almost perfect agreement

| **Reliability Type** | **Observed Agreement (Po)** | **Expected Agreement (Pe)** | **Cohen’s κ** | **p-value** |
| --- | --- | --- | --- | --- |
| Inter-rater (2 raters) | 0.90 | 0.52 | 0.79 | <0.001 |
| Intra-rater (1 rater, n=50) | 0.93 | 0.53 | 0.86 | <0.001 |

Inter-rater agreement was substantial (κ = 0.79), while intra-rater agreement was almost perfect (κ = 0.86), indicating that the assessment of fusion was reliable and reproducible.

1. Landis, J. R., & Koch, G. G. (1977). The measurement of observer agreement for categorical data. *Biometrics*, *33*(1), 159–174.
