# Supplementary material for: Smoking significantly impairs clinical outcome following anterior cervical radiculopathy surgery
Source: Brain Spine. 2026 Apr 1;6:106030. doi: 10.1016/j.bas.2026.106030 (PMC13089042; doi:10.1016/j.bas.2026.106030)
Supplement: Multimedia component 2 [file mmc2.docx]

Appendix 2: the impact of the number of pack years on clinical outcomes

**
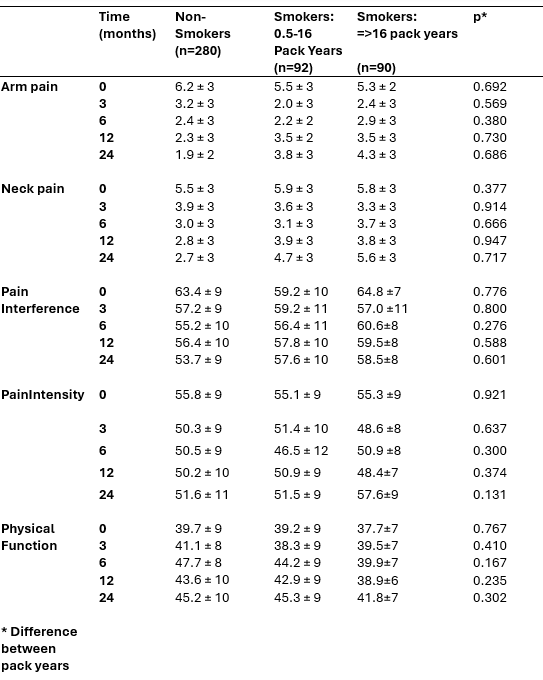
**

An independent t-test was performed.
